# Optical-Beam-Induced Current in InAs/InP Nanowires for Hot-Carrier Photovoltaics

**DOI:** 10.1021/acsaem.2c01208

**Published:** 2022-06-02

**Authors:** Jonatan Fast, Yen-Po Liu, Yang Chen, Lars Samuelson, Adam M. Burke, Heiner Linke, Anders Mikkelsen

**Affiliations:** † NanoLund and Division of Solid State Physics, Lund University, Box 118, Lund 22100, Sweden; ‡ NanoLund and Division of Synchrotron Radiation Research, Lund University, Box 118, Lund 22100, Sweden; § Department of Electrical and Electronic Engineering, Southern University of Science and Technology, Shenzhen, Guangdong 518055, China

**Keywords:** nanowires, optical-beam-induced current, scanning photocurrent microscopy, hot carrier, photovoltaic, InAs, InP

## Abstract

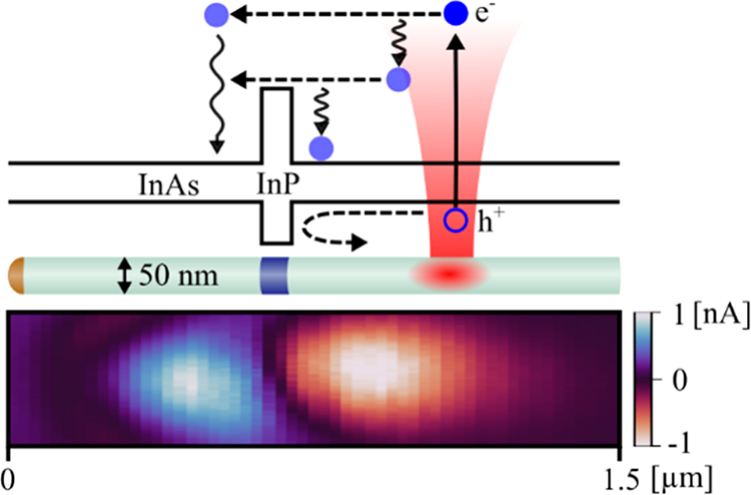

Using the excess energy of charge carriers excited above the band edge (hot carriers) could pave the way for optoelectronic devices, such as photovoltaics exceeding the Shockley–Queisser limit or ultrafast photodetectors. Semiconducting nanowires show promise as a platform for hot-carrier extraction. Proof of principle photovoltaic devices have already been realized based on InAs nanowires, using epitaxially defined InP segments as energy filters that selectively transmit hot electrons. However, it is not yet fully understood how charge-carrier separation, relaxation, and recombination depend on device design and on the location of optical excitation. Here, we introduce the use of an optical-beam-induced current (OBIC) characterization method, employing a laser beam focused close to the diffraction limit and a high precision piezo stage, to study the optoelectric performance of the nanowire device as a function of the position of excitation. The photocurrent response agrees well with modeling based on hot-electron extraction across the InP segment via diffusion. We demonstrate that the device is capable of producing power and estimate the spatial region within which significant hot-electron extraction can take place to be on the order of 300 nm away from the barrier. When comparing to other experiments on similar nanowires, we find good qualitative agreement, confirming the interpretation of the device function, while the extracted diffusion length of hot electrons varies. Careful control of the excitation and device parameters will be important to reach the potentially high device performance theoretically available in these systems.

## Introduction

The extraction of energetic photoexcited carriers, so-called hot carriers, is of high interest for novel optoelectronic applications in particular solar cells^1,2^ as well as photodetectors^1,2^ and light-based neuromorphics with low energy use.^3,4^ Following excitation, hot carriers typically lose their excess kinetic energy within about picoseconds via carrier–phonon, carrier–carrier, and defect interactions, making it a challenge to harvest the carrier energy before it is dissipated.^1^ Semiconducting nanowires (rod-shaped objects with a high length/diameter aspect ratio and diameter on the order of 100 nm and below) are a promising platform to study hot-carrier extraction for several reasons:^5^ nanowires offer flexibility in band engineering due to low restraints on lattice mismatch^6^ and sharp heterointerfaces;^7^ hot carrier temperatures have been observed to increase with shrinking nanowire diameter;^8^ and the quasi 1D geometry simplifies extraction and facilitates control of the position of light excitation.^9,10^

**Figure 1:**
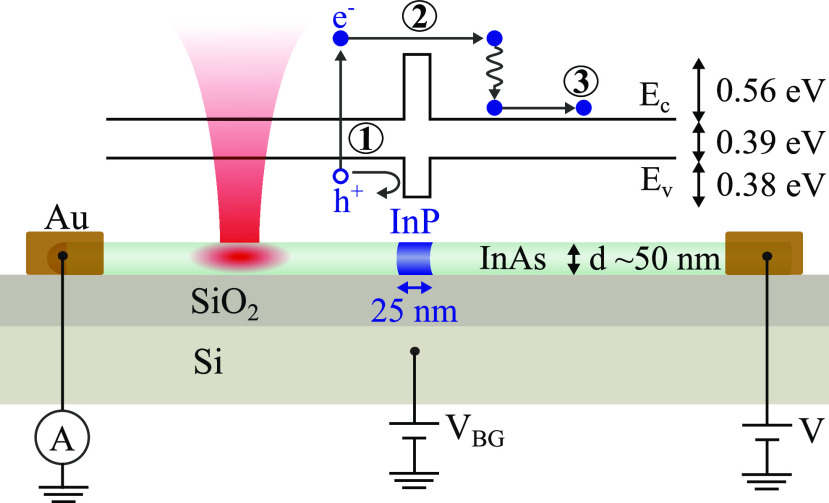
Schematic of the sample structure and principles of the experiment. We use an InAs nanowire with 50 nm diameter. A 25 nm long InP segment located at the nanowire center results in a potential barrier for holes and electrons. The nanowire is deposited on a Si substrate with an insulating 100 nm SiO_2_ layer and contacted at both ends. A laser beam scans across the device, and any current generated is recorded as a function of beam position. Current is thought to be generated according to a mechanism that can be summarized in three steps: (1) excitation of electron–hole pair, where the electron receives a majority of the excess energy due to its lower effective mass; (2) hot electrons use this excess energy to surpass the barrier, resulting in a difference in electrochemical potential across the barrier; and (3) separated charge carriers drift toward the contacts, resulting in a net current.

InAs nanowires with axial, epitaxially defined InP heterostructure segments have been shown to produce a photocurrent under optical illumination, reaching open-circuit voltages above the Shockley–Queisser limit for a corresponding bulk InAs photovoltaic device.^11^ The observed high voltage is believed to be generated by hot electrons diffusing over the energy barrier formed by the large-bandgap InP segment, a process that can be represented in three steps (see Figure 1): (1) optical excitation is focused on one side of the barrier, locally photogenerating electron–hole pairs. Because electrons in InAs have an effective mass that is roughly 10 times smaller than that of holes, electrons receive a majority of the excitation energy.^12^ (2) Energetic (hot) electrons are able to move over the barrier (ballistically or by diffusion), while lower-energy holes are blocked, resulting in charge separation. (3) The charge separation results in a hot-electron photocurrent and a voltage that is not limited by the separation of quasi-Fermi levels (as it would be in a conventional photovoltaic device). A more detailed description of the hot-electron generation would take into account the joint density of states between states in valence and conduction bands that share the same location in k-space. For photon energies considerably higher than the band gap, this will include excitations to secondary conduction band minima, and intervalley scattering can become relevant.^13^

As the diameter of the nanowire is below the diffraction limit for optical light, spatial control of excitation is a challenge that has previously been addressed in two different ways: taking advantage of a naturally occurring wave mode maximum, with geometry- and wavelength-dependent location, when globally illuminating the nanowire,^10^ or with the aid of metal plasmonic elements that enhance light absorption within a small (subwavelength) region.^9^ Both of these approaches require careful alignment of the nanowire and the device components, introducing variations in performance from device to device and making it challenging to systematically tune the location of light excitation. By exposing the nanowire to a focused electron beam, mapping of the electron beam-induced current (EBIC) with high spatial resolution has confirmed that the current originates from charge separation across the InP barrier.^14^ The EBIC technique, however, is the result of a bombardment by high energy electrons and can only to some extent be likened with optical excitation. To study the performance of the device under more realistic operational conditions, we seek a consistent way of optically exciting hot electrons within a small controlled region of the nanowire.

In the present work, we achieve spatial control over optical excitation within single nanowire devices. This allows us to study devices identically fabricated to those used in the previous EBIC study^14^ under more realistic operational conditions. This is done by combining a laser focused close to its diffraction limit and a sample stage that can be moved with high precision using a piezo scanner designed for probe microscopy (see Figure 1). The technique is referred to as optical-beam-induced current (OBIC), also known as light-beam-induced current and scanning photocurrent microscopy (LBIC, SPCM). OBIC has been proven to be a useful technique in other studies on single nanowire devices, although these had no internal heterostructures as in the present case.^15−17^

We observe a pattern of photocurrent generation through the nanowire that agrees well with a theoretical model based on current generation by extraction of hot electrons across the InP barrier (Figure 1). By fitting our model to the data, we extract an effective hot-electron diffusion length on the order of 300 nm. Identifying the illumination locations that optimize the photocurrent produced, we analyze the current–voltage characteristics at these points to show that the device produces power, with a short-circuit current *I*_SC_ = 2.3 (±0.05) nA and an open-circuit voltage *V*_OC_ = 80 (±0.1) mV.

## Experiment

We study InAs nanowires with a diameter of about 50 nm and a length of 2 μm, with an embedded segment of InP located roughly at the center in axial direction. The length of the InP segment is about 25 nm, chosen to be long enough to rule out tunneling but shorter than expected relaxation lengths. It results in a potential barrier of 0.56/0.38 eV in the conduction/valence band, respectively (see Figure 1).^18,19^ Growth is done by chemical beam epitaxy using gold seed particles deposited on an InAs (111)B surface; all segments are of wurtzite crystal structure.^20^ A high crystalline quality with few defects is expected, except for some stacking faults in the growth direction, and the samples are weakly n-type.^20,21^

**Figure 2:**
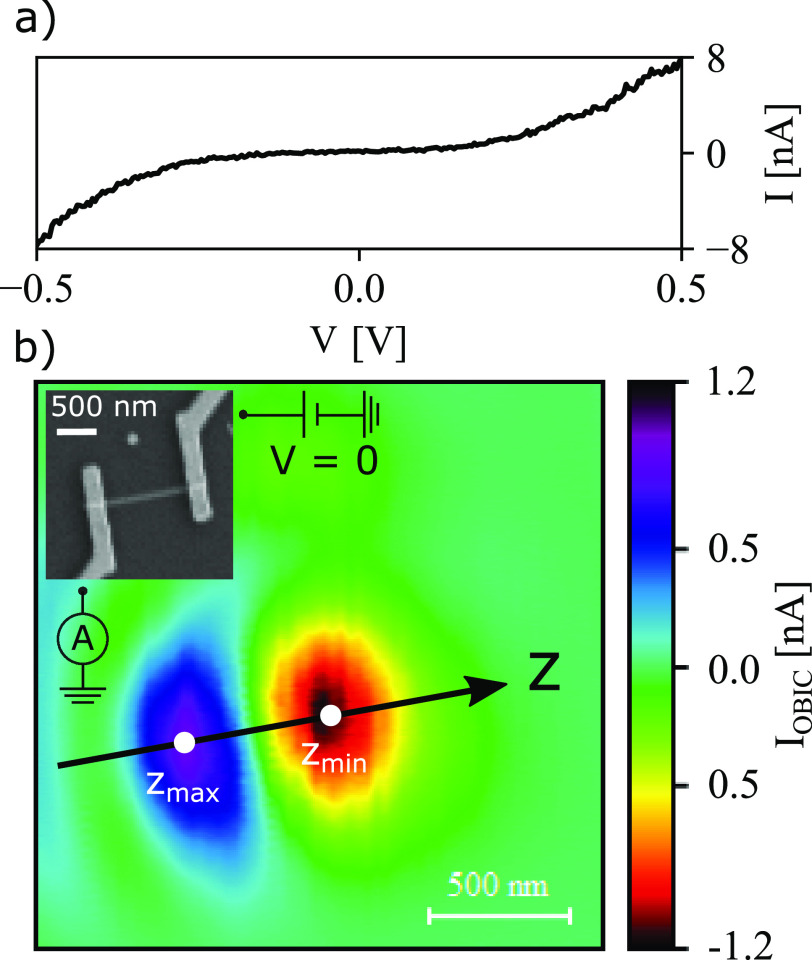
(a) Current–voltage data of the nanowire device in the dark, showing a plateau of low conductance typical for a thermionic barrier. (b) OBIC of the same nanowire device, with a scanning electron micrograph of the device (inset) and circuit schematic showing drain/source contacts. No bias voltage is applied, and all current observed corresponds to generated photocurrent. The OBIC image is oriented in the same way as the scanning electron micrograph, so that the *z*-axis is directed along the nanowire. *z*_max_ and *z*_min_ indicate the points with the highest and lowest current. The InP segment is expected to be located in between these two.

Device fabrication is described in more detail in previous work,^21,22^ and here we give a brief overview (see Figure 1 for device layout). Nanowires are mechanically deposited from the growth substrate to the sample substrate, consisting of n-type Si covered by a 100 nm SiO_2_ layer for electrical insulation. The sample substrate contains predefined gold pads, to which nanowires are contacted via electron beam lithography and metal evaporation of 25 nm Ni followed by 75 nm Au. We use a surface passivation technique^22^ well-known to produce InAs nanowire–metal contacts of Ohmic quality, with the Fermi level pinned roughly 100 meV above the conduction band at the InAs surface.^23,24^ A scanning electron micrograph of a completed device can be seen in the inset of Figure 2b.

For OBIC measurements, the sample is mounted on a piezoelectric motor stage with a Nanonis PD 5 driver, allowing for movements on the order of single Ångströms. All measurements are done at room temperature to resemble the operational conditions of a photovoltaic device. A laser diode, Thorlabs LD785-SEV300, driven by a CLD1010LP controller produces unpolarized, continuous-wave, narrow-bandwidth light with a wavelength of 785 nm (1.58 eV). The laser is focused by an 100× objective lens, Olympus LMPLFLN100×, to achieve a spot size with a full width at half-maximum (fwhm) around 500 nm (see Figure S1 for the beam profile). This yields an irradiance, *E*_e_, of the beam on the order of 4 × 10^3^ W cm^–2^ or a photon flux of 1.6 × 10^22^ s^–1^ cm^2^. In order to create the OBIC map, we first align and orient the device to the laser beam with the help of the 100× object microscope. The sample stage is then rastered within a 6 μm × 6 μm range, while the laser beam is turned on. The rastering is done mainly perpendicular to the nanowire axis and at rates on the order of 100 nm/s (for comparison, hot carriers typically decay significantly faster than 1 ns).^1^ Current through the device is mapped as a function of beam position, resulting in 3D maps. We use a low noise preamplifier, FEMTO DLPCA-200, to record the current, and all biases are applied by a Nanonis SC5 signal controller system.

## Results and Discussion

Initial current–voltage characterization of complete devices (Figure 2a) with no illumination shows a plateau of low transmission, as expected in the presence of a thermionic barrier. Slight asymmetry of the curve is to be expected as the location of the InP barrier is typically not exactly in the center of the nanowire, leading to a slightly different voltage drop on either side of the barrier. Reference measurements of identically fabricated devices based on InAs nanowires without an InP segment and a similar diameter of roughly 50 nm confirm that the contacts are ohmic, showing no sign of a Schottky barrier for electrons (see Figure S4).

OBIC measurements (Figure 2b) reveal two regions of photocurrent, *I*_OBIC_, with opposite polarity along the nanowire, consistent with photocurrent generation by extraction of hot electrons over the InP barrier (Figure 1). The observed current corresponds to electrons moving from the location of excitation to the contact on the opposite side of the barrier. At the same time, holes, being less energetic due to their heavier mass, are less likely to cross the barrier. This results in a net separation of charge carriers and thus a photocurrent based on hot-electron extraction. Following this argument, the location of the InP segment can be identified as the point between OBIC maxima and minima, where *I*_OBIC_ = 0.

**Figure 3:**
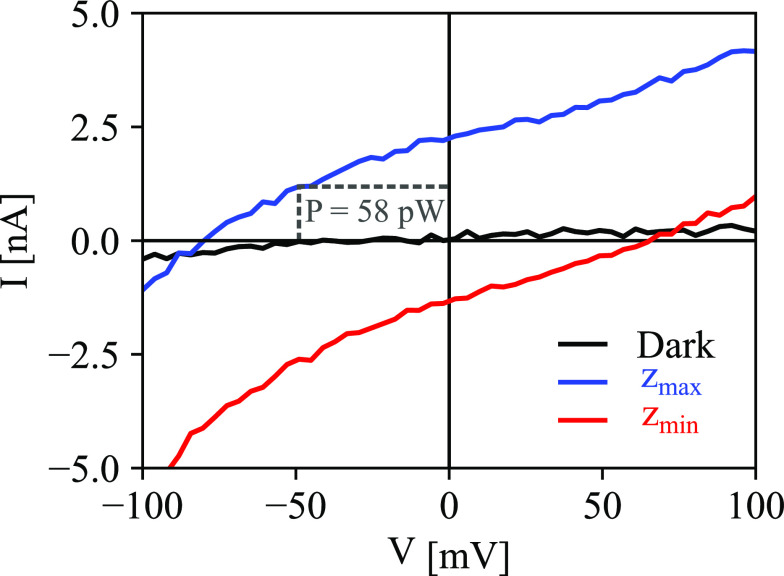
Current–voltage measurement collected under the dark (black), with the laser fixed at the point of maximum *I*_OBIC_/*z*_max_ (blue) and fixed at the point of minimum *I*_OBIC_/*z*_min_ (red). Short-circuit current and open-circuit voltage are extracted for *z*_max_/*I*_SC_ = 2.2 (±0.05) nA, *V*_OC_ = −80 (±0.1) mV, and for *z*_min_/*I*_SC_ = −1.3 (±0.05) nA, *V*_OC_ = 65 (±0.1) mV. Gray box indicates the point of highest power production, *P* = 58 (±4) pW, found at −49 mV.

We performed additional current–voltage characterization (Figure 3) with the laser focused at the extreme points of *I*_OBIC_, *z*_max_, and *z*_min_ (see white marks in Figure 2b). At both points, the device clearly produces power, reaching an open-circuit voltage up to *V*_OC_ = 80 (±0.1) mV, a short-circuit current *I*_SC_ = 2.3 (±0.05) nA, and a maximum power *P* = 58 (±4) pW, with the beam at *z*_max_.

**Figure 4:**
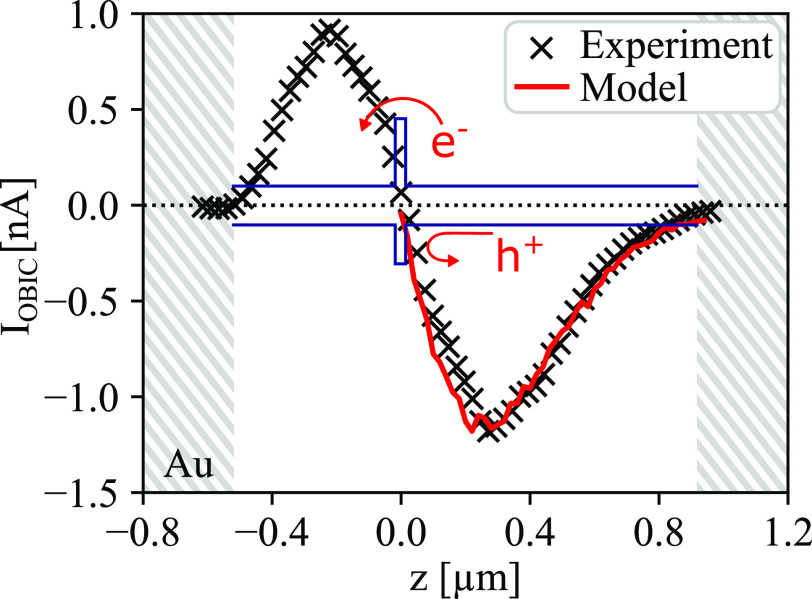
*I*_OBIC_ along the axial direction of the nanowire, indicated by the *z*-axis in Figure 2b. Black crosses are experimental data. The red line current is a fit of the model to the experimental data, yielding a hot-electron diffusion length of *L*_e_ = 280 (±30) nm. Band diagram (blue) is a schematic illustrating the location of the InP barrier (assumed to be at the location where *I*_OBIC_ = 0), and red arrows indicate the direction of carrier transport according to the model.

To better understand the dependence of hot-electron extraction and photocurrent production on generation location, the variation of *I*_OBIC_ with excitation location along the nanowire axis is studied in detail (see Figure 4). The linecut is taken along the axial orientation of the nanowire and at the radial position that gives maximal current, assumed to be the center of the nanowire. The two peaks are separated by approximately 500 nm, the same order as the laser spot size. Thus, the maximum in *I*_OBIC_ occurs when the center of the beam is half of its fwhm away from the barrier, i.e., consistent with the beam fwhm being entirely on one side of, but as close as possible to, the barrier. Of special interest for hot-electron transport is the shape of the decay of the current when moving away from maxima toward the contacts, which can reveal information about the relaxation of hot electrons: the longer the excited carriers need to diffuse to reach the barrier, the more they risk losing their excess energy, and the less likely they are to be transmitted over the barrier and contribute to the current. Because electrons are the majority carriers in the nanowires used here and because a Schottky barrier for holes is expected at the nanowire–contact interface, we expect that holes are not collected at the metal contacts but rather recombine with injected electrons (see Figure S5 for OBIC on InAs nanowires without the InP segment to support this).

We define an effective hot-electron diffusion length, *L*_e_, as the typical distance that a hot electron can diffuse while still having enough energy left to surpass the barrier. In addition to the recombination rate of electron–hole pairs, *L*_e_ depends on several factors such as the initial excess energy and the rates of energy loss via carrier–phonon and carrier–carrier interactions, making it difficult to fully predict. However, we can develop a simple theoretical model of the optical-beam-induced current behavior along the nanowire with *L*_e_ as the only, variable parameter, describing the net effect of all energy decay rates (see SI for details). The model assumes exponential decay in the density of hot electrons as they diffuse away from the point of excitation, such that *I*_OBIC_ ∼ exp(−*z*/*L*_e_) = *D*(*z*), with *z* being the location along the nanowire axial direction, centered at the barrier location. To determine the rate of photogeneration, *G*(*z*), a 3D optical model based on the finite element method and Maxwell’s wave equations, is employed (see SI for details). The current resulting from the beam at a certain location, *z*, is found by integrating the product of *D*(*z*) and *G*(*z*) on the right and left side of the barrier and taking the difference, as detailed in ref (14). The peak value of the calculated current is normalized to the peak of the experimental current. Modeling of the current profile using different values of *L*_e_ is shown in Figure S3 of the SI. It can be seen that, while *L*_e_ does not influence the model profiles close to the barrier, it clearly influences the slope of the OBIC signal decay beyond the maximum of the OBIC signal. Thus, with *L*_e_ as the only free parameter, the model is fitted to experimental data by minimizing the residual sum of squares, giving a best fit for *L*_e_ = 280 (±30) nm (see the red line in Figure 4).

Returning to the experimental data (Figure 4), the signal on the left side of the barrier does not follow the same exponential decay that is seen on the right side. This is likely explained by the barrier being closer to the left than the right contact, so that carrier generation is partially blocked on the left side. No charge separation, and thus net current, is expected when illuminating the metal contacts. The metal contacts will in other words work as an optical mask for the part of the nanowire they cover, preventing optical generation beneath them, cutting the OBIC signal short before the exponential tail is clear. Such optical masking can for example be used to characterize the optical beam, but it precludes using the profile on the left side of the barrier for fitting with our model. We therefore choose to only fit our model on the right side of the barrier.

One can also note that the slope of the decreasing OBIC signal, toward the center of the InP barrier, becomes steeper during the last ∼100 nm (see Figure S3). We propose that this is because the energy of the laser beam is too small to excite electrons inside the InP segment.

We now compare our OBIC results reported to previous EBIC studies on identically fabricated devices based on nanowires from the same growth run (see Figure S6).^14^ The observed curves are qualitatively similar, which is reasonable given that in both cases a hot-electron distribution is created,^25−27^ and the resulting current through the thin InP barrier is measured. Fitting of the EBIC data with the same approach as in this article yields *L*_e_ ≈ 100 nm, lower than observed here. Keeping that in mind, while both methods eventually induce a hot carrier distribution in the few eV range above the conduction band minimum, the excitation created by EBIC (using 3 keV electrons) is very different from the optical excitation during OBIC (using 1–1.5 eV photons in the present study). A quantitative difference such as the one observed here is not surprising and may be due to, e.g., differences in the hot-electron energy distribution and density.

Another noticeable difference compared to the optical beam is the smaller spot size of the electron beam. Given that the InP barrier is smaller than both the electron and optical beam size, the spatial separation between the maximum/minimum currents observed around the barrier reflect the beam size. In the EBIC experiment, the spot size is roughly 60 nm (including the secondary electron cascade), and a peak separation on similar order (around 100 nm) is thus observed. The sharp transition between the peaks of opposite polarity in EBIC makes it clear that charge separation is taking place next to the barrier and not elsewhere along the nanowire. This conclusion can thus be extended to the OBIC data and is an underlying assumption in the interpretation of the results in this work.

We can compare our OBIC and EBIC measurements to optical experiments previously conducted on similar nanowires (60 nm diameter and 70 nm long InP segment) using a pulsed light source and nanofabricated, plasmonic antennas to strongly localize photogeneration of hot carriers.^9^ Under illumination with wavelengths of 954–1240 nm (1.0–1.3 eV), an *L*_e_ on the order of 30–40 nm was estimated in these devices. Variations in *L*_e_ between these three experiments could be due to differences in the energy distribution of carriers and experimental details such as surface composition and carrier density.^28^ In particular, the intensity of the beam is different for the two optical experiments (significantly lower in the present work). An increased density of excited carriers will result in significantly faster carrier–carrier scattering and thus potentially decreased *L*_e_, as seen in III-Vs for electron–electron scattering.^29^ Recent experiments on femto-/picosecond hot-carrier dynamics in InAs nanowires^28^ are consistent with reaching an *L*_e_ of 30–40 nm at high photon densities. The lower photon densities used in this article could well lead to an order of magnitude increase in scattering times and thus *L*_e_.^28,29^ Further, the additional device processing and light localization close to the barrier occurring, when adding a plasmonic antenna,^9^ could also alter the effective loss *L*_e_.

**Figure 5:**
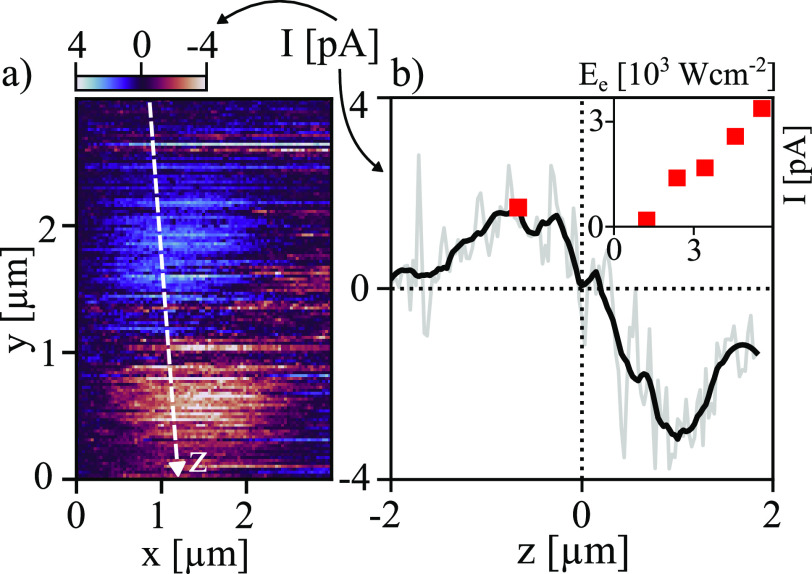
(a) OBIC with 1310 nm (0.95 eV) laser at irradiance *E*_e_ = 3.4 × 10^3^ W cm^–2^, on a similar device as reported inFigure 2 (same growth and fabrication). (b) Current along the nanowire (*z*-axis indicated in a). Gray line indicates actual data and the black line mathematically smoothed data (Savitzky–Golay filter) used to identify peaks more reliably. Red box indicates maximal current at the positive side. Inset shows this point collected at five different irradiances on the same device.

OBIC measurements at long near-infrared wavelengths, low currents, and variable powers were performed with an excitation wavelength of 1310 nm (0.95 eV) on a similar single nanowire device (Figure 5a). The device was fabricated similarly as for previous measurements using wires from the same growth. First, it shows the experimental method applicability across wavelengths and currents levels. Second, it indicates that the mechanism discussed in this paper is consistent across devices and varying excitation conditions. The photocurrent along the nanowire (Figure 5b) has similar shape and polarity as excitations with 780 nm (Figure 4). The current at the maxima (red square) is seen to increase monotonically with irradiance (inset, Figure 5b). These results highlight the versatility of OBIC as a method of studying the photoelectric response in a nanoscale device with both spatial and spectral resolution under various lighting conditions. In future studies on the optimization of hot-carrier extraction in these and similar nanostructures, it is going to be a vital tool.

Using a long near-infrared wavelength for excitation presents some challenges to the resolution of the OBIC data for several reasons; longer wavelengths fundamentally limit the possible spatial resolution achievable by the optics of the setup, and thus the spot size will be larger. In the data, this is seen as a larger separation between the two peaks and a larger width of the peaks (compared to Figure 4). As the excitation energy, 0.95 eV, is at the threshold for directly exciting an electron from the valence band and across the barrier, fewer electrons are expected to be extracted before relaxing, and thus a lower photocurrent is expected, as observed. The lower current leads to a significantly noisier signal (see gray curve in Figure 5b). However, a profile can still be obtained and is enough to derive the power dependence of the current (at a few pA), as seen in the inset of Figure 5b. The present study thus opens up for further exploration of the power/wavelength dependence of the photocurrent, and the experimental technique will be utilized in future studies to study the optimization of power production in single nanowire devices of varying design and illumination conditions. However, this will require prolonged measurement series beyond the scope of the present demonstrational study.

Finally, we rule out the possibility that the OBIC signature of the InP barrier heterostructure originates from charge separation occurring around a barrier formed at the nanowire–metal contact interface. First, it is well established that no Schottky barrier for electrons should exist for the Au/Ni-InAs contacts.^22^ Second, a resulting net charge flow arising from a barrier at the contact interface would be directed in the opposite direction of that observed. Third, OBIC measurements on InAs nanowires without InP segments, but otherwise identical devices, were performed (see Figure S5). Those devices show an OBIC signal with different polarity and shape than seen for the barrier device. The signal from nanowires without a barrier is similar to previous OBIC investigations of nanowires without heterostructures done by others, where a Schottky barrier for holes was deemed the most likely origin of the signal (for further discussion on this, see the SI).^15^

## Conclusions

We have studied the photocurrent resulting from the extraction of hot electrons over an axial potential barrier within a single nanowire by excitation with a laser focused close to the diffraction limit. We show that the device is capable of producing power, and determine the distance from the excitation location to the barrier within which most hot electrons are extracted, the effective hot-electron diffusion length, to be on the order of 300 nm. In previous studies, control of the location of optical absorption within the nanowire relied on sample geometry and external plasmonic elements.^9,10^ In this work, we show that even with its diffraction-limited spatial resolution, OBIC can retrieve important information related to hot-carrier extraction and reveal subwavelength information.

In future work, we envision OBIC to be an important tool in efforts to optimize the performance of nanowire-based hot-carrier photovoltaic devices. The *V*_OC_ observed in the present experiment is small compared to that previously reported for similar devices under optical illumination as well as that theoretically predicted by the Shockley–Queisser detailed balance method.^9,11^ The reason for the lower *V*_OC_ is currently not understood but could be related to a higher presence of surface trap states on the nanowires used here, as this is known to be a great source of variation in the photoconductance of InAs nanowires.^15,30−32^ Passivating the nanowire surfaces to remove oxide states might be crucial for control over this in future devices.^30,32,33^ Additionally, the highest *V*_OC_ is not expected under the same conditions (same location) that yield the maximal *I*_SC_. Future detailed *I*–*V* curves at all locations, and as a function of load resistance, as previously reported InAs nanowires containing quantum dots^34^ could yield further insights into this. The dependence of *L*_e_ and power on the excitation energy of the light source is another interesting point that could be explored. Finally, in order increase the hot-carrier extraction efficiency, nanowires of more complex heterostructure geometries could be created, taking the obtained information on carrier dynamics into account. One such structure would be a two barrier device, with one barrier that extracts hot electrons and one for holes, as further discussed in a recent review article.^5^

## Supporting Information Available

Experimental details, additional OBIC data on InAs nanowires without the InP segment, description of theoretical model and fitting procedure, and comparison between OBIC and EBIC data on similar devices (PDF)
