# Supplementary material for: Optical-Beam-Induced Current in InAs/InP Nanowires for Hot-Carrier Photovoltaics
Source: ACS Appl Energy Mater. 2022 Jun 2;5(6):7728–34. doi: 10.1021/acsaem.2c01208 (PMC9245483; doi:10.1021/acsaem.2c01208)
Supplement: Supplementary file 1 — ae2c01208_si_001.pdf [file ae2c01208_si_001.pdf]

Supporting information

# Optical-beam induced current in InAs/InP nanowires for hot-carrier photovoltaics

*Jonatan Fast‡*[1], Yen-Po Liu‡[2], Yang Chen[1], Lars Samuelson[1,3], Adam M. Burke[1], Heiner Linke[1], Anders Mikkelsen*[2]*

[1]NanoLund and Division of Solid State Physics, Lund University, Box 118, Lund 22100, Sweden

[2]NanoLund and Division of Synchrotron Radiation Research, Lund University, Box 118, Lund 22100, Sweden

[3]Department of Electrical and Electronic Engineering, Southern University of Science and Technology, Shenzhen, Guangdong, China

‡These authors contributed equally.

* Corresponding Authors:

**Jonatan Fast** – *NanoLund & Division of Solid State Physics, Lund University, Box 118, Lund 221 00, Sweden; Email: jonatan.fast@ftf.lth.se*

**Anders Mikkelsen** – *NanoLund & Division of Synchrotron Radiation Research, Lund University, Solvegatan 14, Lund 223 62 Sweden; Email Anders.mikkelsen@ftf.sljus.se*

## Beam profile

The profile of the laser beam at the sample surface is determined by observing the change in photoconductance on a sample of Au electrodes on a light-sensitive InP substrate (Figure S1a). While scanning the laser beam over the two electrodes (along the red line inFigure S1b), the current through the electrodes is recorded (Figure S1c). The differentiated current gives the profile of the beam (Figure S1d, red line). With a Gaussian fit to the differentiated data (Figure S1d, blue line), the full width at half maximum is determined to 0.5 μm.

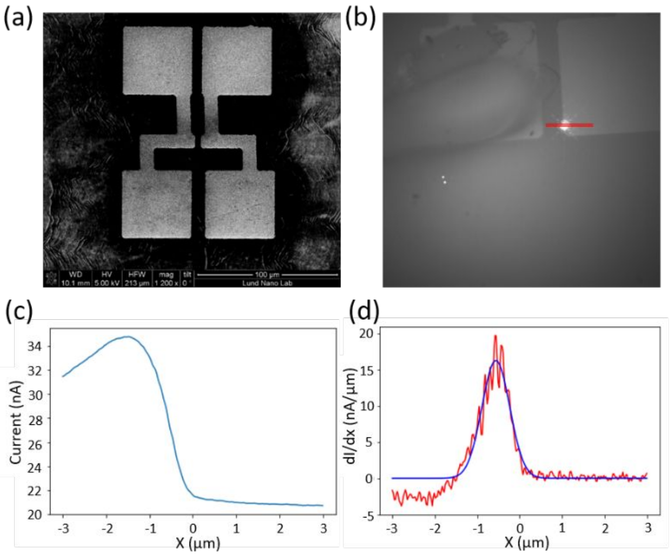


**Figure S1.** a) Scanning electron microscope of Au/InP structure used to measure beam shape. b) The laser beam scanning (along the red line) across one of the gold electrode edge under the optical microscopy, c) and d) current and differentiated current, respectively, recorded as beam sweeps along the red line in b). in d), red line is data and blue is a Gaussian curve fitted to the data with a full width at half maximum of 0.5 μm.

## Modelling of OBIC

Here we describe the approach used to model the OBIC signature along the nanowire and use this to extract a value for $L_e$, seen in Figure 4 of the main text. The model is based on two main components.

First, a one dimensional rate of photo-generated carrier density along the nanowire long axis z, $G_{OBIC}(z)$, is calculated by an optical model. The optical model uses a finite element method (COMSOL Multiphysics) to solve the Maxwell equations with the device geometry parameters from experiment (Figure S2). The InAs nanowire is defined to be 2 μm long, 60 nm in diameter, containing a 25 nm long InP segment in the center (axial direction). It sits on top of a Si substrate covered with a 100 nm thick layer of $SiO_2$, and Au contacts at the nanowire ends are 80 nm thick. The model assumes the incident beam has Gaussian shape, with a waist diameter

of 500 nm a wavelength of 780 nm. The optical absorption, depending on the position of the incident beam, is calculated from the model. By assuming each absorbed photon converts to one electron-hole pairs, a position-dependent carrier generation rate can be calculated as $G_{OBIC}(x,y,z)$ (in unit $m^{-3}s^{-1}$). An integration over the nanowire cross section of $G_{OBIC}(x,y,z)$ leads to the one dimensional optical generated carrier density $G_{OBIC}(z)$ along the nanowire with given geometry and optical parameters. In the end, such a one dimensional carrier generation rate is calculated for a series of beam positions ($z_{beam}$) in the axial (z) direction (simulating the optical beam sweeping along the nanowire).

Second, the probability of a hot electron to be transmitted over the barrier, *D*, is assumed to decrease exponentially the further away, *z*, from the barrier it is generated. This exponential decay is characterized by its effective diffusion length $L_e$:

$$D(z) = e^{-z/L_e} \quad \text{(S1)}$$

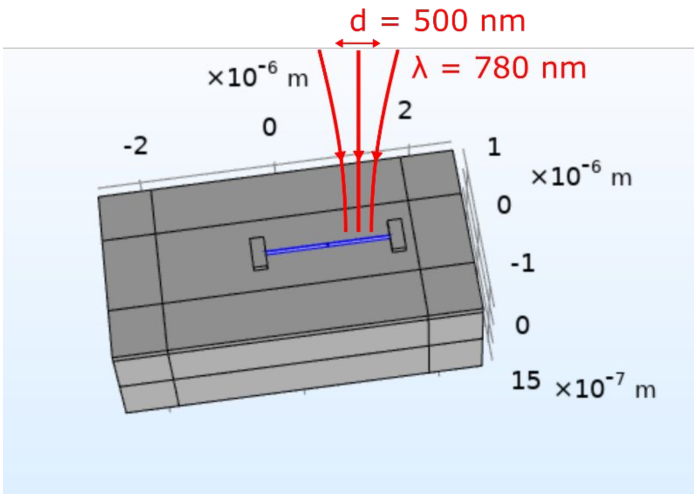


**Figure S2.** Overview of the 3D optical model defined in COMSOL Multiphysics, used to calculate optical absorption (carrier generation rate).

The total current, $I_{\mathrm{OBIC}}$, generated with the laser beam at a certain location, $z_{\mathrm{beam}}$, is then assumed to be proportional to the difference in the integral of the product of $G_{OBIC}(z)$ and $D(z)$.

$$I_{\mathrm{OBIC}}(z_{\mathrm{beam}}) \sim \int_{\mathrm{Left\ contact}}^{\mathrm{barrier}} G_{\mathrm{OBIC}}(z)D(z)dz - \int_{\mathrm{barrier}}^{\mathrm{Right\ contact}} G_{\mathrm{OBIC}}(z)D(z)dz \quad \text{(S2)}$$

Since this is a proportional relation, the calculated $I_{\mathrm{OBIC}}$ must be normalized to match the experimental data. This is done so that the maximum current of experiment and calculated current match.

The same methodology as explained in this section is used and described in previous work with EBIC on similar nanowires, with the only difference being the generation mechanism.[1]

## Fitting

Here we describe the procedure of fitting the calculated $I_{\mathrm{OBIC}}$ to the experimentally measured (see Figure 4 of main text). The only unknown parameter in equation S2 is the effective diffusion length, $L_{\mathrm{e}}$, and the fitting is performed by finding the value $L_e$ that minimizes the residual sum of squares (RSS) between the data and the model:

$$RSS = \sum_{i}^{n} (I_{data}(x_i) - I_{model}(x_i))^2 \quad \text{(S3)}$$

An example of how the fit between data and model looks like for a few different values can be seen in Figure S3. A kink in the model can be noticed around 0.25~0.3 μm which is an artefact of the mesh grid used in the 3D optical model.

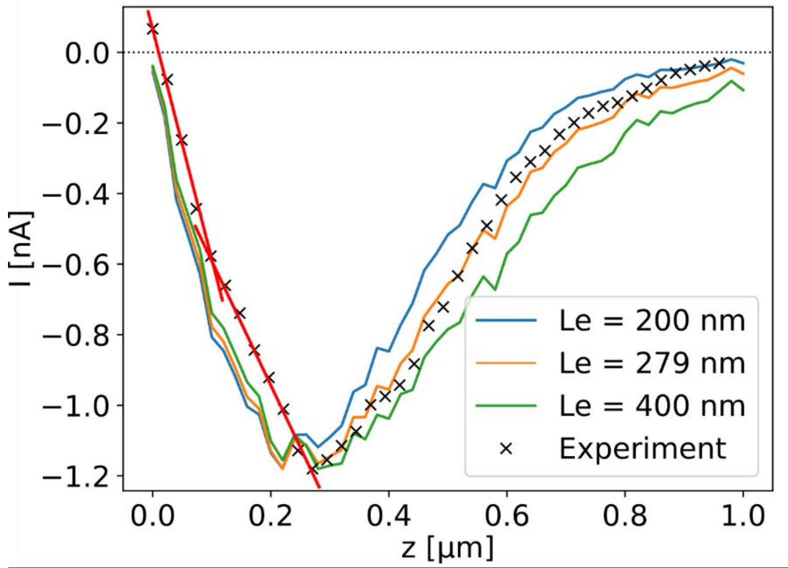


**Figure S3.** The right half of the OBIC current shown in Figure 4 of the main text, experimental values and calculated current for three different values of $L_e$. The two red lines highlight the different slope seen in the regions 0~100 nm and 100~250 nm, believed to originate from lack of absorption in the InP segment.

## Nanowires without InP barrier

For reference, to make clear the impact of the InP barrier on our measurement, we performed OBIC on identically designed devices using "plain" InAs nanowires (Wurtzite crystal structure, 50 nm in diameter), without any InP heterostructure. Current-voltage characterization of such devices are perfectly linear even in a small bias range of ±50 mV, indicating there is no Schottky barrier or similar obstacle for electrons to overcome (Figure S4). If a Schottky barrier for electrons was present, this curve would be expected to take on a shape with a "plateau" of low conductivity around zero bias (similar to that of figure 2 in the main text), since electrons with energy less than a threshold (proportional to the width of such a plateau) would be unable to get over such barrier. As the curve is perfectly linear even in the small bias range of ±50 mV,

it can be concluded that electrons do not face such a barrier traveling through the metal-nanowire contact. This makes us confident the contacting between metal contacts and nanowire is Ohmic also in the device with an InP barrier.

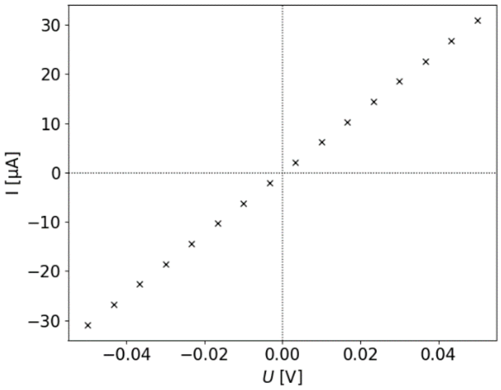


**Figure S4.** Current-voltage sweep of a plain InAs nanowire device.

The nanowire without any barrier shows an OBIC result (Figure S5a) that at first glance appears similar to that of the nanowire with a barrier, with two regions of opposite polarity. The orientation of positive/negative polarity is however opposite to the barrier device, indicating that a net flow of electrons is directed instead towards the contact closest to the location of optical excitation. Similar behavior has been observed during OBIC studies of InAs nanowires by others[2], as well as in PbS nanowires[3] and Si nanowires[4]. It is believed, and predicted by theory,[5] that this signal originates from a Schottky barrier for holes (Figure S5b). In n-type devices where electrons are the majority charge carrier, such a barrier won't have any major effect on bias-induced transport (as made clear by the linearity in Figure S4). However when exciting additional electron hole pairs, as in this experiment, it will impact the photocurrent generated as it can cause charge separation. Out of the portion of excited charge carriers that diffuse towards the metal contact, the holes may get reflected at the Schottky

barrier, while electrons are freely transmitted. This means that there will be a net flow of electrons over the contact closest to the location of generation. Electrons and holes diffusing towards the contact further away from the location of excitation may recombine, but there will be an excess of holes (reflected at the closest contact). These holes can recombine with electrons coming in from the further contact. The mechanism detailed here will result in a current that is the strongest close to the metal contacts, and with opposite polarity compared to that observed in devices with a barrier, thus agreeing well with the observations.

Since the resolution in this experiment is limited to the order of 500 nm, due to the laser spot size, the OBIC of device with/without InP barrier appear more similar than they actually are. The previously reported EBIC experimetns on identical devices however show with higher resolution that in plain InAs devices, the signal originates from the nanowire-metal contact region.[1] For nanowires with a barrier on the other hand, the EBIC signal (Figure S6) clearly originates from the barrier region.

It may seem surprising that no charge separation is observed near the contacts when introducing the InP segment. To understand this, it is important to note that the mechanism for current generation in plain InAs nanowires will essentially be blocked when adding the barrier at the center of the nanowire. Introducing the barrier means that any holes reflected at the contact interface will no longer be able to diffuse towards the opposite contact, being blocked by the barrier. This also tells us that, no matter whether an InP segment is present or not, photoexcited holes that diffuse towards the metal contacts appear to recombine with electrons injected from the contact.

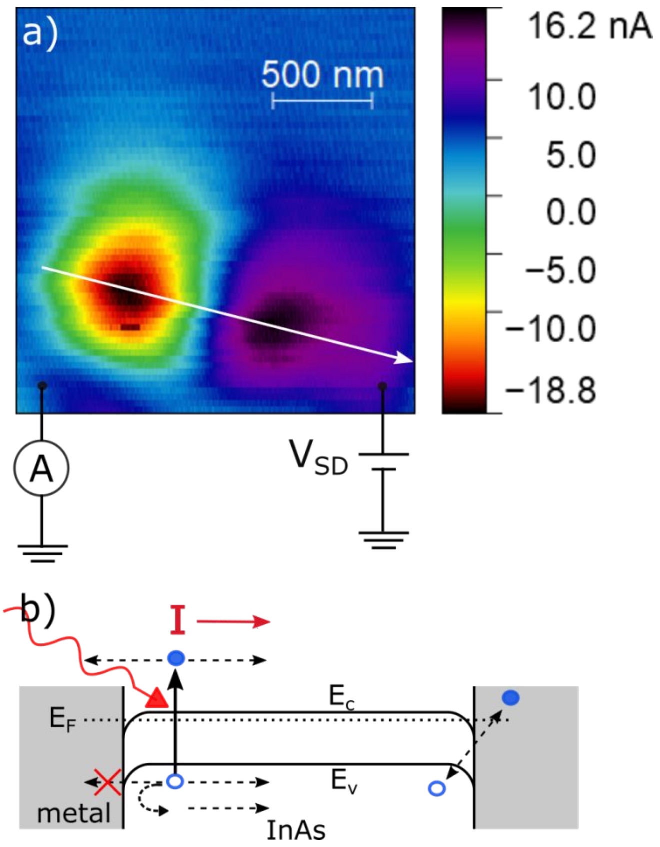


**Figure S5.** a) OBIC of InAs nanowire without any heterostructure, same device as Figure S4. White arrow indicates orientation of nanowire. b) Conceptual sketch of the mechanism expected to explain the origin of the OBIC signal observed. A Schottky barrier for holes is formed at the nanowire-metal contact interface, reflecting holes but transmitting electrons.

## Comparison with EBIC

OBIC data reported in this work is compared with EBIC data from previous work.[1] The devices are fabricated with an identical methodology and design, from nanowires grown in the same growth run.

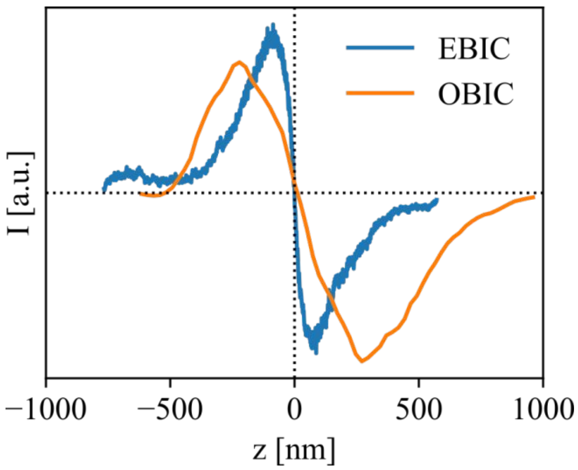


**Figure S6.** EBIC (blue) and OBIC (orange) on identically fabricated devices based on nanowires from the same growth run. EBIC data reproduced from ref[1] with permission from authors.

**Corresponding Author**

* Jonatan Fast – Division of Solid State Physics, Lund University, Sweden; NanoLund, Lund University, Sweden; Email: jonatan.fast@ftf.lth.se

* Anders Mikkelsen – Division of Synchrotron Radiation Research, Lund University, Sweden; NanoLund, Lund University, Sweden; Email: Anders.mikkelsen@sljus.lu.se

**Authors**

Yen-Po Liu – Division of Synchrotron Radiation Research, Lund University, Sweden; NanoLund, Lund University, Sweden

Yang Chen – Division of Solid State Physics, Lund University, Sweden; NanoLund, Lund University, Sweden;

Lars Samuelsson – Division of Solid State Physics, Lund University, Sweden; NanoLund, Lund University, Sweden;

Adam Burke – Division of Solid State Physics, Lund University, Sweden; NanoLund, Lund University, Sweden;

Heiner Linke – Division of Solid State Physics, Lund University, Sweden; NanoLund, Lund University, Sweden;

Anders Mikkelsen – Division of Synchrotron Radiation Research, Lund University, Sweden; NanoLund, Lund University, Sweden

## Author Contributions

‡These authors contributed equally. The manuscript was written through contributions of all authors. All authors have given approval to the final version of the manuscript.

## References

(1) Fast, J.; Barrigon, E.; Kumar, M.; Chen, Y.; Samuelson, L.; Borgström, M.; Gustafsson, A.; Limpert, S.; Burke, A.; Linke, H. Hot-Carrier Separation in Heterostructure Nanowires Observed by Electron-Beam Induced Current. *Nanotechnology* **2020**, *31* (39), 394004. https://doi.org/10.1088/1361-6528/ab9bd7.

(2) Yang, Y.; Peng, X.; Kim, H.-S.; Kim, T.; Jeon, S.; Kang, H. K.; Choi, W.; Song, J.; Doh, Y.-J.; Yu, D. Hot Carrier Trapping Induced Negative Photoconductance in InAs Nanowires toward Novel Nonvolatile Memory. *Nano Lett.* **2015**, *15* (9), 5875–5882. https://doi.org/10.1021/acs.nanolett.5b01962.

(3) Graham, R.; Miller, C.; Oh, E.; Yu, D. Electric Field Dependent Photocurrent Decay Length in Single Lead Sulfide Nanowire Field Effect Transistors. *Nano Lett.* **2011**, *11* (2), 717–722. https://doi.org/10.1021/nl1038456.

(4) Ahn, Y.; Dunning, J.; Park, J. Scanning Photocurrent Imaging and Electronic Band Studies in Silicon Nanowire Field Effect Transistors. *Nano Lett.* **2005**, *5* (7), 1367–1370. https://doi.org/10.1021/nl050631x.

(5) Fu, D.; Zou, J.; Wang, K.; Zhang, R.; Yu, D.; Wu, J. Electrothermal Dynamics of Semiconductor Nanowires under Local Carrier Modulation. *Nano Lett.* **2011**, *11* (9), 3809–3815. https://doi.org/10.1021/nl2018806.
